# Assessing Whether Habitat Suitability Models Can Predict Abundance of an Insect Pest

**DOI:** 10.1002/ece3.74094

**Published:** 2026-07-26

**Authors:** Gengping Zhu, Cesar Rodriguez‐Saona, David W. Crowder

**Affiliations:** ^1^ Department of Entomology Washington State University Pullman Washington USA; ^2^ Department of Entomology Rutgers University Chatsworth New Jersey USA

**Keywords:** blueberry maggot, forecasting, pest distribution, pest management, remote sensing

## Abstract

Predicting pest abundance across landscapes is a major challenge in pest management. Habitat suitability models offer one approach to produce spatially explicit forecasts of pest abundance, but they can often fail to effectively capture heteroscedastic pest distributions. While pests are unlikely to occur in poor habitats, a gradient between low and high pest abundance can occur in suitable habitats, and it is unknown which types of habitat suitability models are most effective in dealing with such wedge‐shaped relationships between abundance and habitat suitability. Here we used six habitat suitability models to assess whether they could predict the abundance of a major native pest of blueberries, the blueberry maggot ( *Rhagoletis mendax* ). We used satellite‐derived images to gather environmental data to build each habitat suitability model, and predictions were compared with *R. mendax* abundance from a 4‐year field survey (2009–2012) in New Jersey, USA. We also modeled how habitat suitability predicts various quantiles of abundance with quantile regressions rather than only the mean. Only two models (BioClim and random forest) detected a positive wedge‐shaped relationship between observed pest abundance and predicted habitat suitability, while four showed a negative relationship. The random forest model performed best in quantile regressions but was poor in transferability; the BioClim model performed poorly in interpolations but was transferrable across spaces. Given the heteroscedastic nature of pest distributions, habitat suitability models may fail to predict pest density unless they capture the gradient of abundance in suitable areas. Different modeling approaches may also be needed for effective interpolation and transferability.

## Graphical abstract

Our research addresses a critical question in pest management: whether habitat suitability models can effectively predict pest abundance across landscapes. We found two models (BioClim and Random Forest) that captured the expected wedge‐shaped relationship, with substantial variation in predictive capacity among models. These results have important implications for pest risk assessment and management.

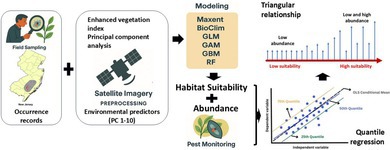

## 1 Introduction

Pest management requires precise spatial data on pest abundance to guide decisions. However, pest monitoring is generally logistically challenging and not always feasible over large scales (Waldock et al. 2022). Even relatively extensive insect monitoring networks often have only one or two traps every 4 to 10 ha, and such trap densities show large variance in captures even in similar areas (Wohleb et al. 2021; Rincon et al. 2024). As a result, spatially explicit estimation of pest abundance is often attempted with statistical models that link data from pest surveys with environmental variables (Rodriguez‐Saona et al. 2018). However, such abundance–environment models often suffer from an inability to predict pest abundance in unsampled localities (Waldock et al. 2022). In addition, data regarding species abundance across a large spatial–temporal context are often unavailable, limiting application of abundance–environment models across broad areas.

**FIGURE 1:**
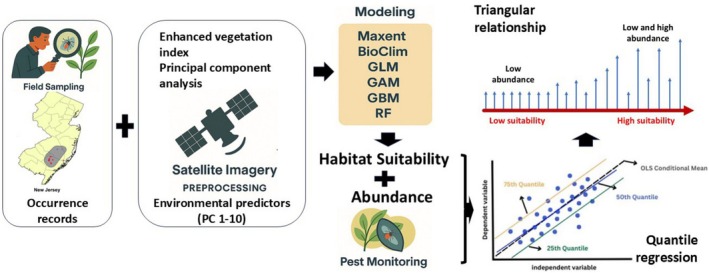
Workflow for modeling habitat suitability and abundance relationships. Six algorithms—(i) Maxent, (ii) BioClim, (iii) generalized linear model (GLM), (iv) generalized additive model (GAM), (v) generalized boosted model (GBM), and (vi) random forest (RF)—were used to estimate habitat suitability based on enhanced vegetation index (EVI) data. These suitability predictions were then compared to observed pest abundance using quantile regression.

Habitat suitability models that relate occurrence data to environmental variables are expected to show a positive correlation between suitability and abundance (Jiménez‐Valverde et al. 2021). Yet, there are many techniques used for building habitat suitability models that may fail to capture heteroscedastic populations. For example, organisms are expected to have a wedge‐shaped or triangular relationship between abundance and habitat suitability (VanDerWal et al. 2009; Tôrres et al. 2012; Acevedo et al. 2017; Monnier‐Corbel et al. 2023). In other words, in habitats that are not suitable, low abundance will always occur, but in habitats that are suitable, there will be a gradient of abundance from low to high (Figure 1). This association occurs because not all habitats that are potentially suitable are ever occupied by the species in question, and several studies report weak or no correlations between pest abundance and predicted habitat suitability (Dallas et al. 2017; Dallas and Hastings 2018; Santini et al. 2019).

Studies to verify associations between abundance and habitat suitability have primarily focused on vertebrates. For example, Maxent models were used to assess relationships between habitat suitability and abundance of 69 rainforest vertebrates (VanDerWal et al. 2009). Other studies have assessed how various types of models deal with the associations. For example, simple and complex habitat suitability models for jaguars ( *Panthera onca* ) were related to observed abundance data, and the BioClim model best captured the wedge‐shaped relationship (Tôrres et al. 2012). Another advantage of this approach is that habitat suitability models can be built using remotely‐sensed variables to capture species habitat requirement with high resolution (Arenas‐Castro et al. 2019). However, while insect pests should exhibit similar wedge‐shaped relationships between abundance and habitat suitability, this has received scant attention despite obvious implications for pest management in agriculture (Weber et al. 2017).

Here, we tested the prediction that abundance of a pest insect would exhibit a wedge‐ shaped response with habitat suitability models. We focused on the blueberry maggot ( *Rhagoletis mendax* ), a major native pest of commercially grown blueberries in the USA. We used six model algorithms to construct habitat suitability predictions, and employed quantile regression analysis to assess the associations between habitat suitability and observed pest abundance (Tôrres et al. 2012; Acevedo et al. 2017). Specifically, we used quantile regression to explore how habitat suitability predicts various quantiles of abundance, rather than only the mean. We considered how model fit was affected by habitat suitability predictions themselves (i.e., variance across models) as opposed to inherent variance in abundance data. Our goals were to (1) verify whether a wedge‐shaped relationship between abundance and habitat suitability occurs for a major insect pest, (2) assess whether different types of modeling approaches affected inferences, and (3) determine whether such models may be used to guide pest risk assessment.

## 2 Materials and Methods

### 2.1 Study System and Pest Sampling

**FIGURE 2:**
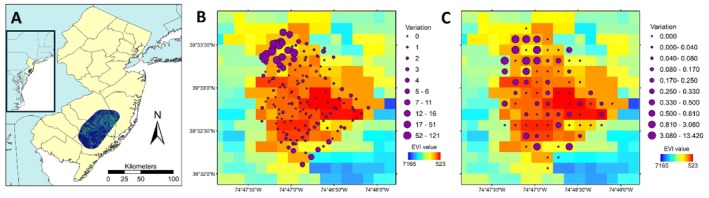
(A) Map of the study sites, showing the location in the Northeast United States and sites in New Jersey. Depicted are pest abundances at various sites, showing the (B) coefficient of variation of *R. mendax* population density across 4 years' survey and the (C) coefficient variation of population density within grid cell, where they were averaged to match environmental variables used in models. The background colors show the environmental vegetation index (EVI) captured on June 2010.

*Rhagoletis mendax* is native to North America and is a pest of highbush and lowbush blueberries in the Northeast United States (Rodriguez‐Saona et al. 2015, 2018). *Rhagoletis mendax* larvae feed on the internal tissues of blueberry, causing the fruit to be unmarketable, and there is low industry tolerance for infested fruit (Rodriguez‐Saona et al. 2015). The occurrence of *R. mendax* in the United States and Canada is regularly monitored to prevent introductions and limit outbreaks (Vincent et al. 2016; Rodriguez‐Saona et al. 2018). We gathered data on *R. mendax* abundance from 9 blueberry farms in southern New Jersey, United States, from 2009 to 2012 (Figure 2A); 2 farms were located in Burlington country and 7 in Atlantic county (Rodriguez‐Saona et al. 2018). Adult *R. mendax* were monitored with Pherocon AM‐baited traps (Trécé Inc., Adair, OK) at a density of 1 trap per ha. A total of 531 traps were placed across all sites in the upper part of the canopy, with trap locations marked using a hand‐held GPS device (Rodriguez‐Saona et al. 2018). Traps were monitored twice a week starting the 1st week of June until the 2nd week of September each year, with lures changed every 15 days.

### 2.2 Insect Abundance and Environmental Data

To capture the average spatial dynamics of *R. mendax* over the 4 years, we aggregated these monitoring data within grid cells of 250 m size (Figure 2B). This data aggregation was done to provide a standardized density measure that matched the resolution of the satellite imagery used in the habitat suitability models; in each grid cell (250 × 250 m) we calculated the average *R. mendax* adult abundance over the four sampling years (Figure 2C). Green areas often indicate adequate soil moisture, which is favorable conditions for *R. mendax* , where dry and low‐green areas tend to reduce their survival. Green habitat can be identified from satellite imagery at fine scales using metrics such as the enhanced vegetation index (EVI). Here EVI images were selected for habitat modeling because it captures vegetation vigor at fine spatial scales. In addition, because *R. mendax* is typically more abundant near forested habitats than non‐blueberry crops (Rodriguez‐Saona et al. 2018), EVI provides an ecologically meaningful proxy of near forested habitat. While our monitoring data reflected a granular dataset at a 250 m resolution, given the relatively limited geographic scope (2 counties), coarse climatic or other variables would not be effective in building habitat suitability models.

We gathered EVI from the moderate resolution imaging spectroradiometer (MODIS) to match the spatial and temporal scale of insect sampling. This provides a rich characterization of landscapes that were expected to be positively associated with *R. mendax* abundance. Sixteen‐day EVI composites were obtained for June to August from 2009 to 2012 at a 250 m resolution, corresponding to the timing of our *R. mendax* field sampling. Rather than using highly correlated raw EVI images, we used a principal component analysis with ten components to fit our habitat suitability models; these principal components captured ~86% of variance and are independent of each other, which are suitable for fitting distributional models. The field trap abundance data, species occurrence records, and principal component datasets are available from the Open Science Framework at https://doi.org/10.17605/OSF.IO/5QJH8. Overview of the workflow for modeling habitat suitability and abundance relationships was provided in Figure 1.

### 2.3 Species Distributional Models

Six habitat suitability models were built around sites (Figure 2A): (i) maximum entropy (Maxent), (ii) bioclimatic envelope (BioClim), (iii) generalized linear model (GLM), (iv) generalized additive model (GAM), (v) generalized boosted model (GBM), and (vi) random forest (RF). BioClim is a presence‐only model, while the other models use statistics or machine learning to relate environmental data to occurrence, and pseudo‐absence data (GAM, GBM, GLM, RF) or background data (i.e., Maxent) (Sillero 2011), and each type of model has been used to test the wedge‐shaped relationship (Tôrres et al. 2012). Habitat models were built on accessible areas delimited by buffering minimum convex polygon of southern training points at 5 km. For the Maxent model, we used the random method to select 10,000 pseudo‐background records and modified the default settings with minimum AIC criteria (Muscarella et al. 2014). For GLM, GAM, GBM, and RF models, pseudo‐absence records were selected at five times the number of presence records within the southern buffered area. We used the *ntbox* platform with default settings to fit the BioClim model (Osorio‐Olvera, Lira‐Noriega, et al. 2020) and the *biomod2* platform to fit the GLM, GAM, GBM, and RF models (Thuiller 2003).

We spatially split data for training and testing, with 70% of the South sampling data (i.e., 145 points; Figure 2) for model training. The remaining 30% in the South were used to test the effectiveness of models in interpolation, the ability to predict occurrence within the same area models were trained on. For this, we plotted 1‐omission error against the proportion of areas predicted to be present. In contrast, the 27 grid cells in the North sampling area were used to validate model transferability, or whether the model trained in the South effectively predicted occurrence in the North. To assess model transferability, we calculated omission error of spatial independent testing points (i.e., the North 27 grid cells) at the minimum, 5th, and 10th training thresholds. These thresholds account for errors in records used in model calibration by allowing 0%, 5% or 10% of training data to be omitted from the binary distributional estimates, and which are suitable for presence‐only distributional models (Peterson et al. 2011).

### 2.4 Quantifying Abundance‐Suitability Correlation

We hypothesized habitat suitability values would be positively associated with the abundance values in each grid cell. Quantile regressions are a powerful tool to quantify the effectiveness of models in capturing wedge‐shaped relationship between abundance and habitat suitability (Acevedo et al. 2017; Jiménez‐Valverde et al. 2021). Quantile regression estimates multiple rates of change (slopes) across different intervals of the response variable (i.e., quantiles), providing a more complete picture of relationships between variables that are often missed by regression methods based on a single mean slope (Cade and Noon 2003). We log +1 transformed abundance data for *R. mendax* in each grid cell to compare with habitat suitability values that were rescaled from 0 to 1 from each of the six habitat suitability models. We fit linear quantile regressions at the 50th, 60th, 70th, 80th, 90th, 95th, 97th, and 99th percentiles of abundance; such delimitations spanned the range of abundance that may be damaging to crops and focus more on high abundance values. All data points were used to fit quantile regressions.

Quantile regressions were performed with each habitat suitability model, where we used R1 to evaluate goodness‐of‐fit of the quantile regressions at each percentile, which was the weighted sum of absolute residuals (Acevedo et al. 2017). To assess the effectiveness of each model in capturing the wedge‐shaped relationship, we examined slope values in quantile regressions: slopes > 0 in any quantile indicate a positive relationship between suitability and abundance, and more slopes > 0 across all quantiles would signify the model effectively captured the wedge‐shaped relationship. We plotted slopes and R1 across percentiles to compare such relationships detected in these models. We used the *quantreg* package in R to perform quantile regressions.

## 3 Results

**FIGURE 3:**
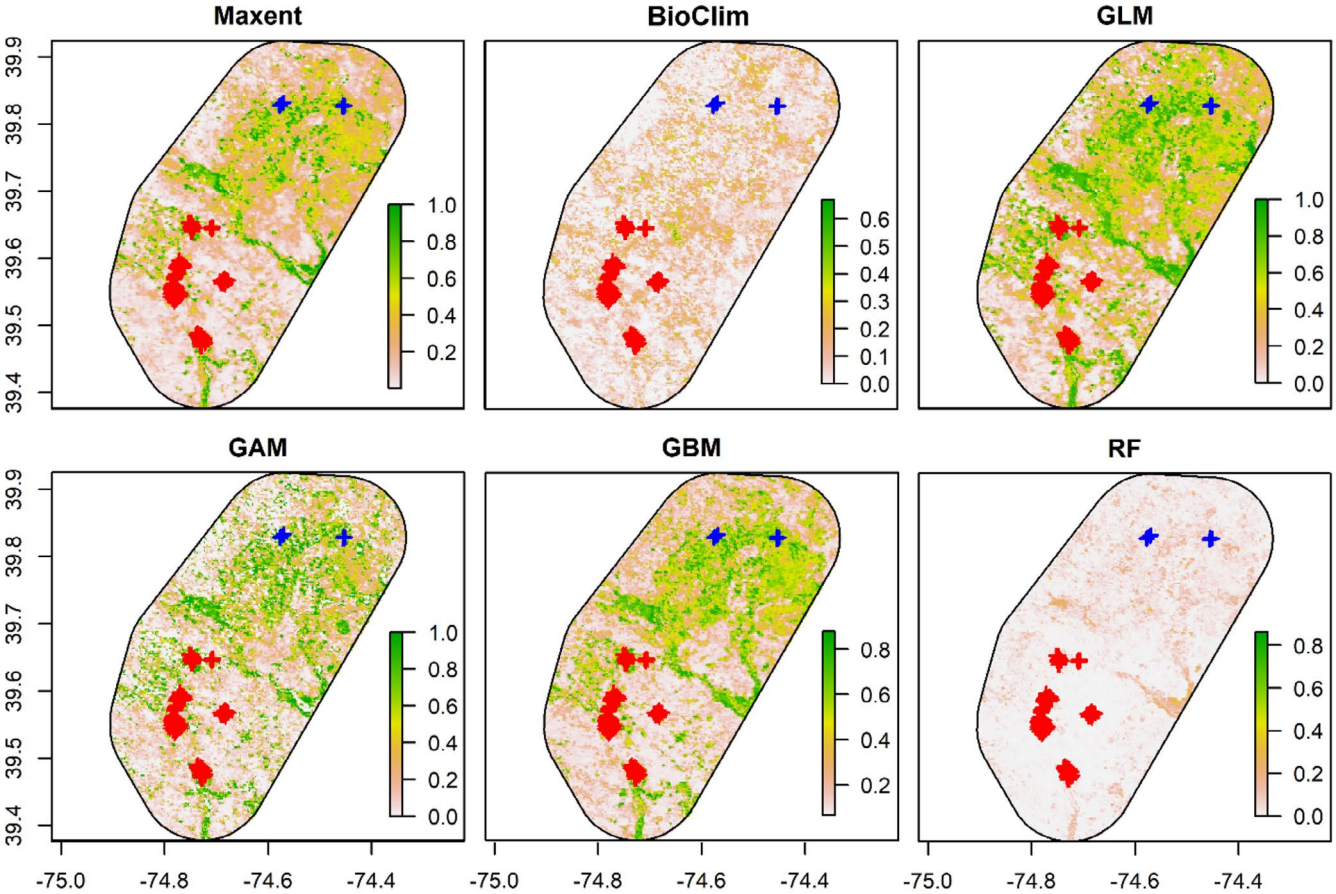
Habitat suitability predictions of *R. mendax* with six models: (i) maximum entropy (Maxent), (ii) bioclimatic envelope (BioClim), (iii) generalized linear (GLM), (iv) generalized additive (GAM), (v) generalized boosted (GBM), and (vi) random forest (RF). Red crosses denote the input training points, and blue crosses denote the independent testing points.

**FIGURE 4:**
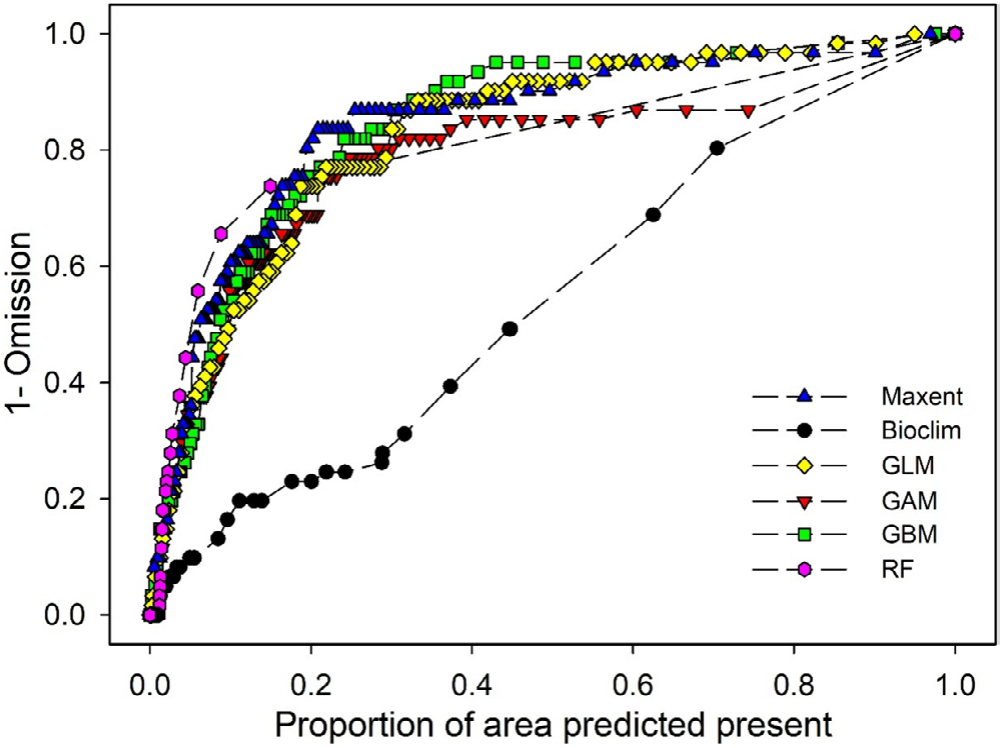
ROC curves show omission errors from interpolations with six models (abbreviations as in Figure 2 legend). A slope of 1 indicates model predictions were not better than random.

**FIGURE 5:**
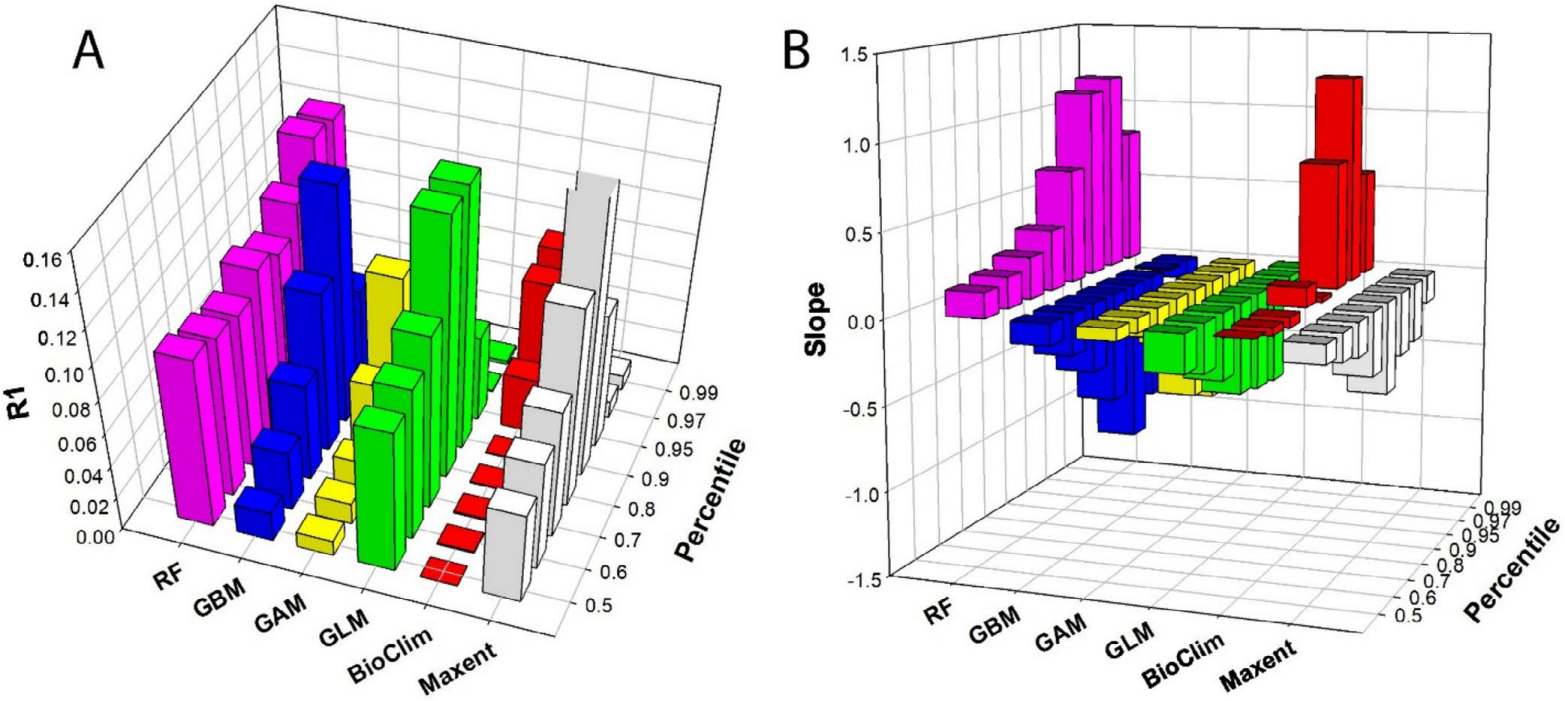
The values of (A) R1 and (B) slope across multiple percentiles in quantile regressions for the six habitat suitability models (abbreviations as in Figure 2 legend). Slopes > 0 indicate a positive relationship between habitat suitability and abundance in a quantile, and multiple slopes > 0 across quantiles indicate an effective model.

The spatial predictions of *R. mendax* habitat suitability models largely clustered into two groups: the BioClim and RF models identified limited suitable habitats in the study area, with RF identified as less than BioClim (Figure 3); whereas the Maxent, GLM, GAM, and GBM models predicted extensive suitable areas across the study area (Figure 3). However, the models differed considerably in validations of interpolation and transferability (Figure 4, Table 1) and in analyses of the wedge‐shaped relationship (Figure 5).

**TABLE 1:** Omission errors in transferability evaluations for six models: (i) Maximum entropy (Maxent), (ii) bioclimatic envelope (BioClim), (iii) generalized linear model (GLM), (iv) generalized additive model (GAM), (v) generalized boosted model (GBM), and (vi) random forest (RF). Models were evaluated at the minimum training presence (MTP) or the 5th or 10th training thresholds. The area occupied by *R. mendax* that was correctly predicted with each model is listed as a proportion.

Threshold	Metrics	Maxent	BioClim	GLM	GAM	GBM	RF
MTP	Area predicted	0.95	0.71	0.88	0.46	0.34	0.01
	No of success	0	14	0	22	23	0
	No of fail	27	13	27	5	4	27
	*p*	NA	0.97	NA	0.00	0.00	NA
5th training	Area predicted	0.64	0.71	0.62	0.21	0.24	0.00
	No of success	25	14	26	14	16	0
	No of fail	2	13	1	13	11	27
	*p*	0.00	0.97	0.00	0.00	0.00	NA
10th training	Area predicted	0.53	0.71	0.44	0.18	0.19	0.00
	No of success	25	14	26	14	11	0
	No of fail	2	13	1	13	16	27
	*p*	0.00	0.97	0.00	0.00	0.00	NA

Abbreviation: NA, not applicable.

The BioClim model performed poorly in tests of interpolation, its ROC curve slope was close to 1 (Figure 4), indicating its predictions were not better than random; whereas all other models' ROC curves stepped far away from the slope 1 (Figure 4), indicating they had strong performance and predicted well across the training area, these ROC curves nonetheless were not parallel, suggesting that the relative performance of these models changed across thresholds.

When considering the transferability of models trained on Southern grid cells (i.e., the red training points in Figure 3) to predict occurrence of Northern grid cells (i.e., the blue testing points in Figure 3), the best three models were the BioClim, GAM, and GBM (Table 1); these three models performed well at all three training thresholds but especially at the minimum‐training thresholds (Table 1). In contrast, Maxent and GLM models performed poorly in transferability at the minimum‐training threshold but well at the 5th and 10th training thresholds, with two points were failing in the Maxent model and one point failing in the GLM model (Table 1). RF models failed to capture all independent testing points at all training thresholds (Table 1).

The RF model was the best in quantile regressions, with R1 values consistently above 0.1 (Figure 5A). The Maxent, GLM, GAM, and GBM models were similar, with R1 values increasing along the 0.5 to 0.9 quantiles and then decreasing (Figure 5A). The BioClim model was poor across all quantiles, with an R1 near 0 that only rose after the 0.9 quantile (Figure 5A). However, only the RF and BioClim models detected a wedge‐shaped relationship between observed abundance and predicted habitat suitability, with slopes greater than 0 observed in all high quantiles (*p* < 0.001, Figure 5). In contrast, the Maxent, GAM, GLM, and GBM models all showed a neutral or even negative relationship between observed abundance and predicted habitat suitability (Figure 5B).

## 4 Discussion

The wedge‐shaped relationship between abundance and habitat suitability is generally expected as organisms cannot thrive (or exist) in unsuitable habitats but can exist at a gradient from low to high abundance in suitable habitats (VanDerWal et al. 2009; Tôrres et al. 2012; Acevedo et al. 2017; de la Fuente et al. 2021). However, this relationship has largely just been tested for vertebrates of conservation concern, with studies on insects being uncommon (Weber et al. 2017; Contreras‐Díaz et al. 2022). Here, we used various models to investigate the wedge‐shaped relationship between observed abundance and predicted habitat suitability for a blueberry pest, *R. mendax* . We found *R. mendax* habitat suitability was not consistently positively related to its abundance, with only two of the six models demonstrating the expected wedge‐shaped relationship. The gradient of abundance occurring in a suitable habitat might depend on how well its physiological and ecological requirements are met at that site in a given year (VanDerWal et al. 2009). A relatively low abundance in potentially suitable habitats could also be simply due to insect management or a lack of nearby overwintering sites (Rodriguez‐Saona et al. 2018).

Our habitat suitability models varied in their predictability performance and in the analyses of the wedge‐shaped relationship (i.e., quantile regression). In model performance, the interpolation reflects the ability of a model to predict unsampled points within the area a model was trained on, while transferability reflects the ability of a model trained on one area to predict values in another area (Heikkinen et al. 2012). Ideally, one or a few models will perform well in both interpolation and transferability, and we observed this with the GLM, GAM, and GBM models. In contrast, the best performing model in quantile regressions (RF) was poor in transferability, and the BioClim model was poor across assessments. Our results reinforce the notion that different models widely vary in their sensitivity to spatial and temporal variability (Qiao et al. 2015). As the best models for interpolation may differ from transferability or quantile regressions, modelers should exercise great caution when predicting abundance.

The wedge‐shaped relationship between *R. mendax* abundance and habitat suitability has implications for pest monitoring and management. Identifying areas of low suitability could guide producers on fields where they can avoid intensive management, or farms where they can exercise more caution before taking management actions (Illán et al. 2022). Although identifying potentially highly suitable areas does not guarantee these areas will have pest outbreaks, these predictions may also guide producers on areas that warrant more intensive sampling or management actions. At the same time, areas that have high predicted suitability, but low pest abundances warrant further consideration as to the mechanisms driving these patterns. Disentangling environmental factors that account for low density, as opposed to factors such as movement or management, may identify factors to adopt into pest management programs. Incorporating landscape factors known to affect pest abundance (Rodriguez‐Saona et al. 2018) into habitat suitability models is another method to aid in making effective predictions.

Despite our results, investigating wedge‐shaped relationships in insects warrants caution, as there are limitations on habitat suitability models and constraints on accurately collecting species abundance values (Hortal et al. 2010; Rincon et al. 2024). For example, there is often large uncertainty in predictions of habitat suitability models, with sources related to the selection of predictors or pseudo‐absence records, or model algorithm and setting (Zhu and Peterson 2017), and this variability can confound the interpretation of the relationships (Wiens et al. 2009). For example, we used high‐resolution EVI as it would capture fine‐scale vegetation greenness, which often reflects adequate soil moisture favorable to *R. mendax* . Other variables (e.g., soil type, temperature, and precipitation) could improve habitat predictions; high‐resolution data for these variables nonetheless were unavailable in our study area.

Methods used to aggregate abundance values across grid cells might also affect the analysis; care should be taken to consider how trap data from a monitoring network are aggregated (Wohleb et al. 2021; Illán et al. 2022). For example, we built habitat suitability models using high‐resolution EVI data, and the integration of remote sensing data into models can present challenges due to matching data collected at far different scales (Montoya et al. 2009; Arenas‐Castro et al. 2019). Our habitat suitability model predictions were generated at a scale appropriate for management of *R. mendax* , although this scale may not be the most appropriate for assessing the wedge‐shaped relationship that may occur at continental scales (Ferrer‐Ferrando et al. 2025), where climate is the primary factor dominating species (insect) distribution (Guisan and Thuiller 2005; Hortal et al. 2010).

Despite our predictions, we found strong associations between *R. mendax* habitat suitability and abundance with two of our models, and the strength of the relationships varied across model algorithms, validation thresholds, and abundance quantiles. Further, different models varied in performance in interpolation versus transferability, suggesting a “one‐size‐fits‐all” approach to abundance prediction is unlikely to be effective (Tôrres et al. 2012; Acevedo et al. 2017). However, our results are promising given that they show we can use granular satellite imagery to build habitat suitability models that can estimate abundance patterns at scales relevant to management (Osorio‐Olvera, Yañez‐Arenas, et al. 2020). As habitat suitability models can be projected with environmental and management scenarios, they could also be widely used to promote sustainable agriculture (Osorio‐Olvera, Yañez‐Arenas, et al. 2020).

## 5 Conclusion

Our study provides a novel test of whether habitat suitability models can effectively predict pest abundances across variable landscapes. We found that habitat suitability models do not consistently predict insect pest abundance and that their effectiveness depends strongly on model algorithm and intended application. Only two of six models identified the expected abundance–suitability pattern, and these models showed clear trade‐offs between predicting pest density and transferring predictions across space. This highlights that no single model is optimal for all applications. From a pest‐management perspective, habitat suitability models are best suited for identifying low‐risk areas and prioritizing monitoring rather than forecasting outbreak severity. Effective use of these models requires aligning model choice with management goals, spatial scale, and validation strategy. Incorporating additional ecological and landscape drivers of pest abundance will be critical for improving risk assessment and supporting more targeted, efficient pest management.

## Author Contributions

**Gengping Zhu:** conceptualization (equal), data curation (equal), formal analysis (equal), investigation (equal), methodology (equal), validation (equal), visualization (equal), writing – original draft (equal), writing – review and editing (equal). **Cesar Rodriguez‐Saona:** funding acquisition (equal), project administration (equal), validation (equal), visualization (equal), writing – review and editing (equal). **David W. Crowder:** funding acquisition (equal), project administration (equal), validation (equal), writing – original draft (equal), writing – review and editing (equal).

## Funding

This research was supported by USDA Hatch (1014754), Multistate Research Hatch (NE2001), and USDA APHIS (1A.0264.01).

## Conflicts of Interest

The authors declare no conflicts of interest.

## Data Availability

The field trap abundance data, species occurrence records, and principal component datasets are available from the Open Science Framework at https://doi.org/10.17605/OSF.IO/5QJH8.
